# Supplementary material for: Exogenous hormones influence Brassica napus leaf cuticular wax deposition and cuticle function
Source: PeerJ. 2020 Jun 4;8:e9264. doi: 10.7717/peerj.9264 (PMC7276146; doi:10.7717/peerj.9264)
Supplement: Table S1 [file peerj-08-9264-s001.docx]

**Supplementary Table S1** **List of primers used for qRT-PCR.**

| Gene name | Forward primer 5’→3’ sequence | Reverse primer 5’→3’ sequence | PCR size (bp) |
| --- | --- | --- | --- |
| *BnCER1-1/2* | AACAAGGTATGGCTGATGGGAGAA | GAATTATTAAAGCCGGTGGAGTATGAT | 147 |
| *BnCER3* | GGAGGAGTGGTTCATATGCTGGA | CCGTGTCTCATGGCTGCTTC | 101 |
| *BnCER4-1/2* | CGACAGAAATGGAGGCCGTAAG | TGCCACCCTTAATATCTTCTCCACA | 116 |
| *BnCER6-1/2* | GTTGAGCTTCTTCGGATGGGTC | GTGCGTGGCTTGGACATGAA | 137 |
| *BnMAH1-1/2* | ATGGCGATGATAGGCTTACCTGA | AAGCATCCCGAGGACAGGC | 129 |
| *BnPDF1-2* | GGCTAAGTTTGCTTCCATCATCACC | TGTCCCACTTGACCTCTCGCA | 112 |
| *BnERF2* | TAGGAGGAGAGGCCGAGTTGTG | CGGACGACGGCGATGACG | 156 |
| *BnPR1* | AACGAGAAGGCTAACTATAACCACGA | TTCCACCATTGTTACACCTCGCTT | 112 |
| *BnActin7* | GTGACAATGGAACTGGAATGGTGA | GTGCCTAGGACGACCAACAATACTC | 92 |
