# Supplementary material for: Exogenous hormones influence Brassica napus leaf cuticular wax deposition and cuticle function
Source: PeerJ. 2020 Jun 4;8:e9264. doi: 10.7717/peerj.9264 (PMC7276146; doi:10.7717/peerj.9264)
Supplement: Table S2 — Notes. ACL = (∑Cn × n)∕∑Cn, where Cn is the relative abundance of compound homolog with n carbons; for total ACL, the new Cn was calculated with the following equations. C27=C26ol+C26al+C26ic+C27an+C27sl+C27on;C28=C27ol+C27al+C27ic+ C28an+C28sl+C28on;C29=C28ol+C28al+C28ic+C29an+C29sl+C29on;C30= C29ol+C29al+C29ic+C30an+C30sl+C30on;C31=C30ol+C30al+C30ic+C31an+ C2731+C31on;C33=C32ol+C32al+C32ic+C33an+C33sl+C33on. [file peerj-08-9264-s002.docx]

**Supplementary Table S2 Effects of hormone treatments on average chain length (ACL) of cuticular wax compounds on leaves of *Brassica napus* plants.**

| Cultivar | Treatments | Fatty acids | Aldehydes | Alkanes | Secondary alcohols | Primary  alcohols | Total |
| --- | --- | --- | --- | --- | --- | --- | --- |
| ZS9 | Control | 29.08±0.12 | 29.15±0.17 | 29.31±0.07 | 29.01±0.03 | 28.18±0.08 | 29.40±0.04 |
|  | SA | 27.53±2.71 | 29.92±0.02 | 29.40±0.02 | 28.96±0.02 | 27.82±0.07 | 29.43±0.04 |
|  | MeJA | 28.97±0.09 | 29.94±0.02 | 29.40±0.04 | 28.87±0.06 | 28.01±0.04 | 29.48±0.05 |
|  | ACC | 29.18±0.26 | 29.95±003 | 29.43±0.03 | 28.96±0.02 | 28.20±0.08 | 29.59±0.04 |
| YY19 | Control | 29.40±0.02 | 29.37±0.14 | 29.72±0.08 | 28.97±0.08 | 27.75±0.15 | 29.49±0.04 |
|  | SA | 29.03±033 | 29.50±0.38 | 29.32±0.07 | 29.01±0.01 | 28.21±0.10 | 29.46±0.08 |
|  | MeJA | 29.04±0.18 | 29.65±0.32 | 29.25±0.10 | 28.93±0.09 | 28.12±0.08 | 29.44±0.04 |
|  | ACC | 29.25±0.04 | 29.34±0.11 | 29.07±0.02 | 29.02±0.01 | 28.17±0.06 | 29.45±0.07 |

**Notes.**

ACL= (∑C_n_× n)/ ∑C_n_, where C_n_ is the relative abundance of compound homolog with n carbons;

For total ACL, the new Cn was calculated with the following equations. C27= C26 ol + C26 al + C26 ic + C27 an + C27 sl + C27 on; C28= C27 ol + C27 al + C27 ic + C28 an + C28 sl + C28 on; C29= C28 ol + C28 al + C28 ic + C29 an + C29 sl + C29 on; C30= C29 ol + C29 al + C29 ic + C30 an + C30 sl + C30 on; C31= C30 ol + C30 al + C30 ic + C31 an + C27 31 + C31on; C33= C32 ol + C32 al + C32 ic + C33 an + C33 sl + C33 on.
