# Supplementary figures and images for: Exogenous hormones influence Brassica napus leaf cuticular wax deposition and cuticle function
Source: PeerJ. 2020 Jun 4;8:e9264. doi: 10.7717/peerj.9264 (PMC7276146; doi:10.7717/peerj.9264)

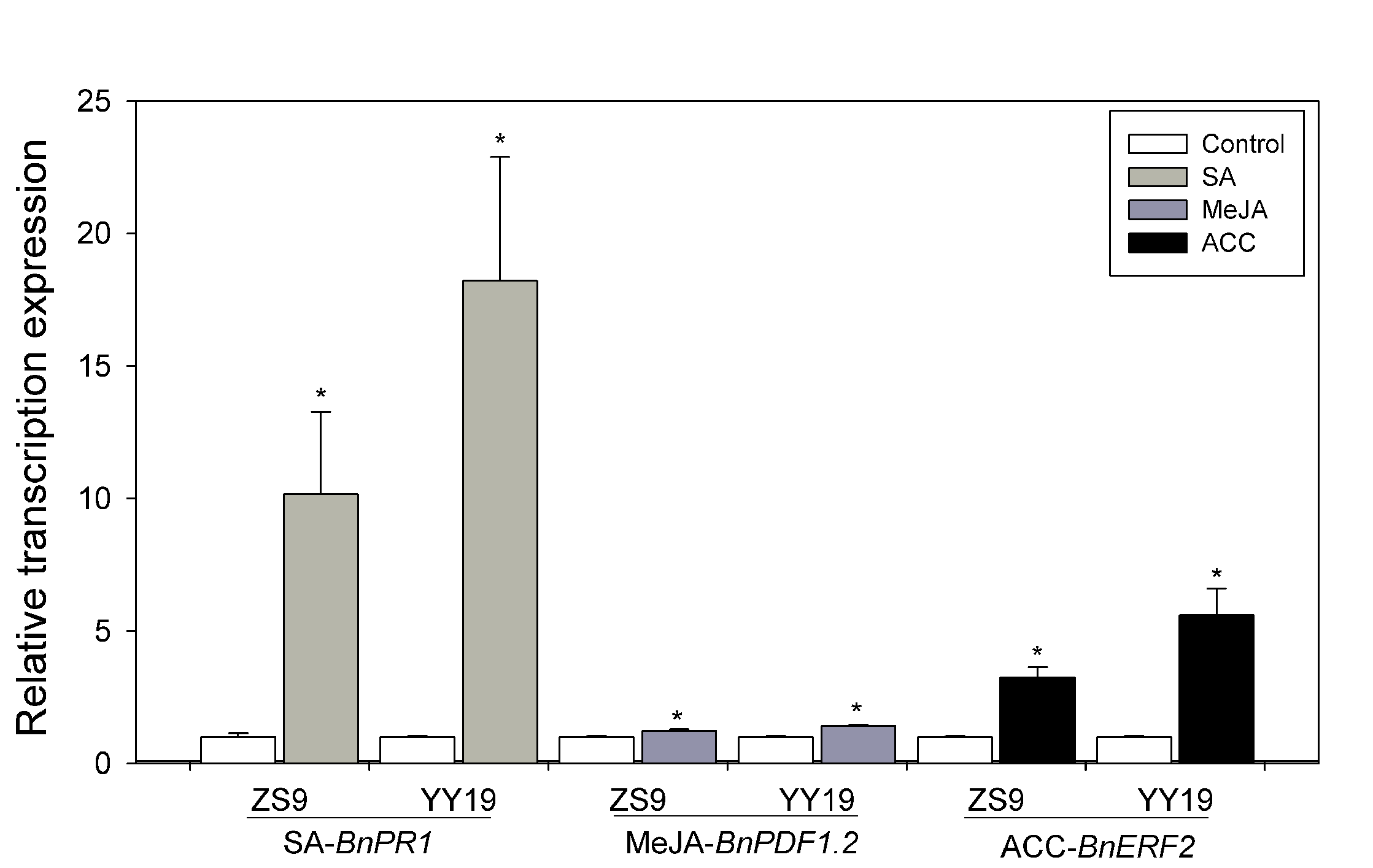

Supplement: Figure S1 — Plants were treated with 0.2 mM SA, 0.1 mM MeJA or 0.2 mM ACC for 21 d. The data represented the average of three biological replicates plus/minus standard deviation. Bars with asterisk represent significance at P < 0.05 according to student T test when compared with the control for each gene. [file peerj-08-9264-s003.png]
